# On Using Maximum *a Posteriori* Probability Based on a Bayesian Model for Oscillometric Blood Pressure Estimation

**DOI:** 10.3390/s131013609

**Published:** 2013-10-10

**Authors:** Soojeong Lee, Gwanggil Jeon, Gangseong Lee

**Affiliations:** 1 Department of Electronics and Computer Engineering, Hanyang University, 222 Wangsimni-ro, Seongdong-gu, Seoul 133-791, Korea; 2 Department of Embedded Systems Engineering, Incheon National University, 119 Academy-ro, Yeonsu-gu, Incheon 406-772, Korea; 3 School of General Education, Kwangwoon University, 447-1 Wolgye-dong, Nowon-gu, Seoul 139-701, Korea; E-Mail: gslee@kw.ac.kr

**Keywords:** oscillometric blood pressure estimation, systolic and diastolic ratios, Bayesian model, maximum amplitude algorithm

## Abstract

The maximum amplitude algorithm (MAA) is generally utilized in the estimation of the pressure values, and it uses heuristically obtained ratios of systolic and diastolic oscillometric amplitude to the mean arterial pressure (known as systolic and diastolic ratios) in order to estimate the systolic and diastolic pressures. This paper proposes a Bayesian model to estimate the systolic and diastolic ratios. These ratios are an improvement over the single fixed systolic and diastolic ratios used in the algorithms that are available in the literature. The proposed method shows lower mean difference (MD) with standard deviation (SD) compared to the MAA for both SBP and DBP consistently in all the five measurements.

## 1. Introduction

There is an increasing need to offer health care devices within the homes of senior patients. This has led to an increasing demand on home blood pressure monitors. Oscillometric measurements have recently gained popularity and are used in blood pressure (BP) monitors, which are now readily available on the market [1–9]. Although vendors of oscillometric BP monitors rarely disclose their algorithms, and the determination of oscillometric systolic blood pressure (SBP) and diastolic blood pressure (DBP) values has been declared controversial [1], the maximum amplitude algorithm (MAA) is one of the most popular algorithms for estimation of arterial blood pressure (ABP) using the oscillometric measurement [2–4]. The MAA approximates the mean blood pressure as the cuff pressure (CP) at which the maximum oscillation in amplitude occurs and then linearly relates the SBP and DBP to this mean pressure using heuristically obtained ratios [2,3,5]. These ratios are utilized to determine the time points at which the cuff pressure (CP) coincides with the systolic and diastolic pressures, respectively [5]. Even though estimation of the ABP is possible employing oscillometric blood pressure estimation, it is subject to various errors since the systolic and diastolic blood pressures or the systolic and diastolic ratios are determined only using quasi-empirical methods [2,4,10]. Moreover, there has been almost no study that tries to estimate the theoretical relationship between the ratios [5]. Currently, the fixed ratios of SBP and DBP are used to determine the SBP and DBP estimates based on the maximum amplitude (MA) obtained through MAA. Although the SBP and DBP ratios (SBPR and DBPR) in the conventional method are assumed to be fixed, this assumption is not valid [5,6,10–12]. For example, Moraes *et al.* [11] suggested that the SBPR and DBPR vary in relation to the SBP, DBP, and mean blood pressure. More recently, the error mechanisms of the fixed-ratio for estimating SBP and DBP was found by Liu *et al.* [10]. Specifically, the fixed ratio may be viewed as a value dependent on the measurements obtained for a specified group of subjects by minimizing the mean absolute error relative to reference auscultatory measurements. If the ratios obtained from one group are utilized for another group, one would not be able to acquire reliable blood pressure estimates. Thus, the MAA derived by a single group-based ratio is not adequate to accurately determine the SBP and DBP because they are subject to significant continuous variability over time [13]. For this reason, it is necessary to develop a methodology that can accurately measure blood pressure based on the oscillometric measurement. Therefore, to obtain a more generalized ratio, this paper proposes a methodology to determine the ratios for systolic and diastolic blood pressures using a Bayesian model for individual subjects. This ratio is an improvement over the single fixed SBPR and DBPR used in the algorithms which are available in the literature.

The organization of this paper is as follows. In Section 2, the data used in the study is described, and the Bayesian model principles and the conventional methodology used in this paper are explained. In Section 3, the experimental results and discussion obtained from the proposed method are presented. In Section 4, conclusions drawn based on the results are presented.

## 2. Methods

The proposed methodology assumes generalized Gaussian distribution [14] which includes not only the Gaussian distribution but also the Laplacian distribution to find more efficient parametric modeling for the *a posteriori* distribution of the SBPR and DBPR, which are used to estimate the SBP and DBP for each individual subject. The methodology also assumes that the ratios are random variables unlike the existing approaches in the literature where they are considered to be deterministic. This paper chooses the value of the likelihood function that maximizes the *a posteriori* probability. An equally likely *a prior* is used that creates a likelihood function using the blood pressure values obtained through the MAA algorithm for each prior. It then chooses the likelihood value that maximizes the *a posteriori* probability obtained using Bayes' model. As a result, the mean difference (MD) and the standard deviation (SD) [15] of the SBP and DBP estimate obtained with the SBPR and DBPR using the Bayesian model were compared with the MD and SD of the estimates obtained using the conventional MAA method.

### 2.1. Conventional MAA Concepts

**Figure 1.**
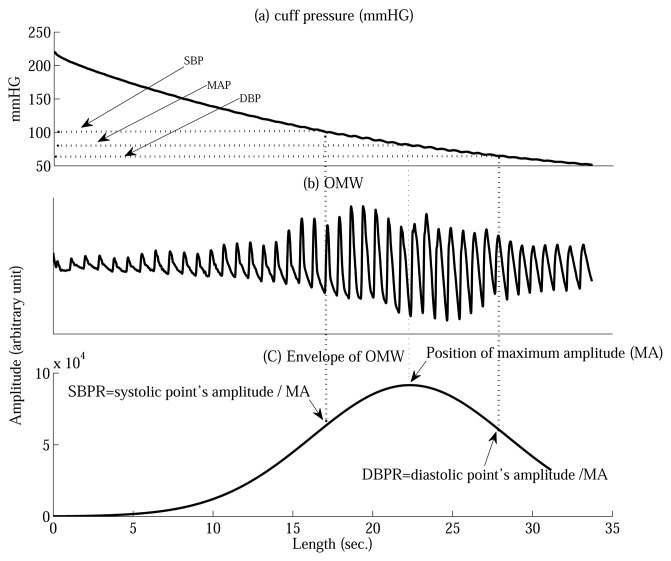
The concept of maximum amplitude algorithm (MAA). (**a**) cuff pressure (CP); (**b**) oscillometric wave (OMW); (**c**) Envelope of oscillometric wave (OMW).

Based on the oscillometric BP envelope, the MAA is widely used to estimate the SBP and DBP, which utilizes SBPR and DBPR to find the points which correspond to SBP and DBP. The amplitude of the maximum point is multiplied by the fixed SBPR and DBPR obtained experimentally [2,3,5].

(1) $${\hat{S}}_{i,j}^{a}=m_{i,j}\times {\hat{r}}^{s}$$

(2) $${\hat{d}}_{i,j}^{a}=m_{i,j}\times {\hat{r}}^{d}$$

where ${\hat{S}}_{i,j}^{a}$ and ${\hat{d}}_{i,j}^{a}$ are the oscillometric amplitudes corresponding to the SBP and DBP, respectively, *m_i,j_* is the maximum oscillometric amplitude (MA), ${\hat{r}}^{s}$ and ${\hat{r}}^{d}$ are the fixed SBPR and DBPR, and *i* = 1,…, *N* and *j* = 1,…, *M*; *N* and *M* denote the number of subjects and the number of measurements per subject. Thus, the oscillometric amplitudes corresponding to the SBP and DBP are mapped back to the deflation curve obtaining the SBP and DBP values in mmHG as shown in Figure 1.

### 2.2. Blood Pressure Estimation Using Bayesian Model

**Figure 2.**
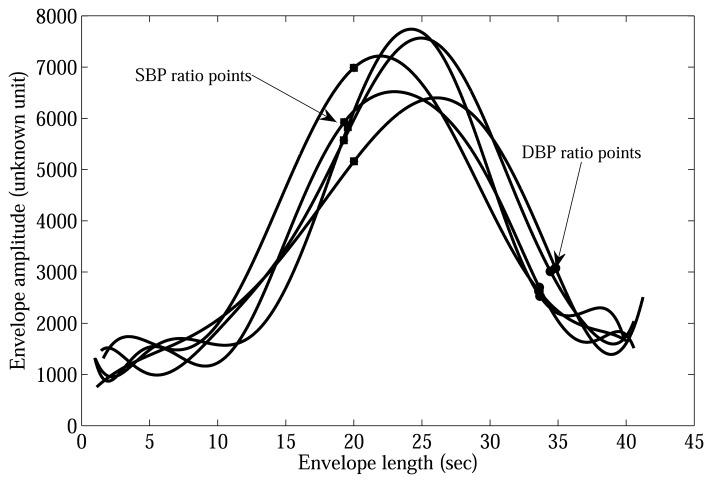
Systolic blood pressure (SBP) and diastolic blood pressure (DBP) ratio-points are obtained by the auscultatory nurse measured with respect to one subject (five measurements); the SBP ratios (0.91, 0.81, 0.97, 0.74, 0.75); the DBP ratios (0.41,0.48, 0.36, 0.40, 0.33).

**Figure 3.**
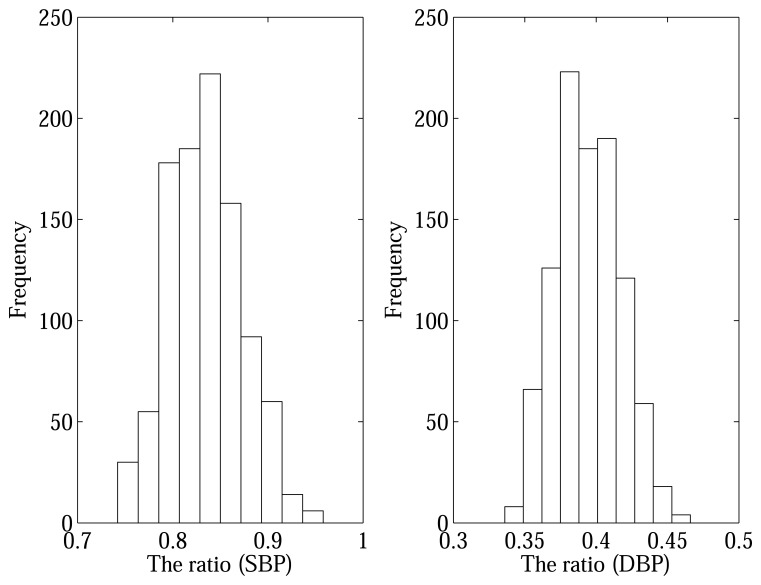
The distribution of pseudo ratios for the SBP and DBP using the nonparametric bootstrap (NPB).

For any *i^th^* subject and any *j^th^* measurement, the systolic and diastolic ratios are defined as follows:

(3) $${\hat{r}}_{i,j}^{s}=\frac{{\hat{S}}_{i,j}^{a}}{m_{i,j}}$$

(4) $${\hat{r}}_{i,j}^{d}=\frac{{\hat{d}}_{i,j}^{a}}{m_{i,j}}$$

where ${\hat{r}}_{i,j}^{s}$ and ${\hat{r}}_{i,j}^{d}$ are the estimated systolic and diastolic ratios respectively, ${\hat{S}}_{i,j}^{a}$ and ${\hat{d}}_{i,j}^{a}$ are the oscillometric amplitude corresponding to the SBP and DBP, respectively, while *m_i,j_* is the maximum oscillometric amplitude (MA) and *i* = 1,…, *N* and *j* = 1,…, *M*; *N* and *M* denote the number of subjects and the number of measurements per subject. It is also conjectured at this point that the systolic and diastolic ratios at the current measurement have no dependence on any of the previous measurements and is only dependent on the physiological status *h* of the person, as shown in Figure 2, which shows the systolic and diastolic points obtained by the auscultatory nurse measurements for one subject. It is also assumed that the physiological status of the person has not changed drastically from measurement, to measurement and the *a prior* probability of the ratios $P({\hat{r}}_{i,j}|h)$ is uniformly distributed between the known the *a priori* minimum and the maximum values. Furthermore, it has been observed by authors that the *a posteriori* distribution of the ratios is Gaussian, concentrated about the mean ${\hat{r}}_{i\mu }.$ As the number of measurements increases with respect to one subject, the probability density function (PDF) of ${\hat{r}}_{i,j}^{s}$ and ${\hat{r}}_{i,j}^{d}$ becomes normal and more concentrated about ${\hat{r}}_{i\mu }$ (shown in Figure 3) as observed using a nonparametric bootstrap (NPB) technique [16] and confirmed through a normality test as presented in [17]. The NPB method is most useful technique where we do not know the sampling distribution. As the distribution of the pseudo SBP ratios using the NBP approximates the distribution of the original SBP five ratios, we use to check normality of the SBP ratio's distribution [17]. Specifically, the distribution of the pseudo SBP ratio in Figure 3a are obtained from each individual subject employing the NPB algorithms because we have only five measurements for each subject. A similar procedure is followed for the pseudo DBP ratio as shown in Figure 3b. The fundamental concept of the NPB technique is to offer a large number of independent bootstrap ratios by resampling the original five ratios **r** = (*r*_1_, *r*_2_,…, *r_m_*) of *m* measurements at random from a unknown probability distribution *F*. A bootstrap resamples ${\text{r}}_{1}^{*}(=x_{1}^{*},x_{2}^{*},\ldots ,x_{m}^{*}),\ldots {\text{r}}_{B}^{*}(=x_{1}^{*},x_{2}^{*},\ldots ,x_{m}^{*})$ are acquired by sampling *m* time drawn randomly with replacement from the original sample **r** with elements occurring zero, once or multiple times, where *m* denotes an original ratio size (=5) and *B* denotes a number of resamples [4,16]. Based on Efron, *et al.* [16], we use *B* = 1,000 [4].

Let **cr***_s_* and **cr***_d_* be the vectors of the possible candidates for the SBPR and DBPR.

(5) $${\text{cr}}_{s}=[\alpha_{1},\alpha_{2},\cdots ,\alpha_{K}]$$

(6) $${\text{cr}}_{d}=[\beta_{1},\beta_{2},\cdots ,\beta_{K}]$$

where **cr***_s_* and **cr***_d_* denote the vectors of the possible candidates for the SBPR and DBPR, *K* is determined *a priori*, where *K* is the number of candidate ratios. In this work, *K* = 31, (*α*_1_ = 0.65 to *α_k_* = 0.95 and *β*_1_ = 0.30 to *β_k_* = 0.60) for the SBP and DBP [11], respectively, in increments of 0.01.

(7) $${\text{pp}}_{s}=[\gamma_{1},\gamma_{2},\cdots ,\gamma_{K}]$$

(8) $${\text{pp}}_{d}=[\delta_{1},\delta_{2},\cdots ,\delta_{K}]$$

where **pp***_s_* and **pp***_d_* denote the *a priori* probability (PP) vectors; the elements of the vector are 1/*K* = 0.032. Due to the lack of any *a priori* information, equal *a priori* probability is assigned to all the candidate ratios. Please note that **cr***_s_*_(_*_i,j_*_)_ = **cr***_s_*, **cr***_d_*_(_*_i,j_*_)_ = **cr***_d_*, **pp***_s_*_(_*_i,j_*_)_ = **pp***_s_* and **pp***_d_*_(_*_i,j_*_)_ = **pp***_d_* for all *i* and *j*. For more details on the basic concept of Bayes rule is given in appendix.

The *a posterior* probability (POP) for every *l*, *l* = 1, · · ·, *K* is

(9) $$p\left(\text{cr}{\left(l\right)}_{s(i,j)}|{\hat{y}}_{s(i,j)}\right)=\frac{\text{pp}{\left(l\right)}_{s(i,j)}f\left({\hat{y}}_{s(i,j)}|\text{cr}{\left(l\right)}_{s(i,j)}\right)}{\sum_{l=1}^{K}\text{pp}{\left(l\right)}_{s(i,j)}f\left({\hat{y}}_{s(i,j)}|\text{cr}{\left(l\right)}_{s(i,j)}\right)}$$

(10) $$p\left(\text{cr}{\left(l\right)}_{d(i,j)}|{\hat{y}}_{d(i,j)}\right)=\frac{\text{pp}{\left(l\right)}_{d(i,j)}f\left({\hat{y}}_{d(i,j)}|\text{cr}{\left(l\right)}_{d(i,j)}\right)}{\sum_{l=1}^{K}\text{pp}{\left(l\right)}_{d(i,j)}f\left({\hat{y}}_{d(i,j)}|\text{cr}{\left(l\right)}_{d(i,j)}\right)}$$

where **pp**(*l*)*_s_*_(_*_i,j_*_)_ and **pp**(*l*)*_d_*_(_*_i,j_*_)_, denote the *a priori* probability for the *l^th^* candidate ratio, and $f({\hat{y}}_{s(i,j)}|\text{cr}{\left(l\right)}_{s(i,j)})$ and $f({\hat{y}}_{d(i,j)}|\text{cr}{\left(l\right)}_{d(i,j)})$ denote the likelihood for the SBP and DBP at the chosen ratio, respectively The conditional measurement distribution of ${\hat{y}}_{s(i,j)}|\text{cr}{\left(l\right)}_{s(i,j)}$ and ${\hat{y}}_{d(i,j)}|\text{cr}{\left(l\right)}_{d(i,j)}$ are normal with a known mean and variance. Their densities are given by

(11) $$f({\hat{y}}_{s(i,j)}|\text{cr}{\left(l\right)}_{s(i,j)})=\frac{1}{\sqrt{2\pi }\sigma }\exp^{-\frac{1}{2\sigma^{2}}{\left({\hat{y}}_{s(i,j)}-\text{cr}{\left(l\right)}_{s(i,j)}\right)}^{2}}$$

(12) $$f({\hat{y}}_{d(i,j)}|\text{cr}{\left(l\right)}_{d(i,j)})=\frac{1}{\sqrt{2\pi }\sigma }\exp^{-\frac{1}{2\sigma^{2}}{\left({\hat{y}}_{d(i,j)}-\text{cr}{\left(l\right)}_{d(i,j)}\right)}^{2}}$$

where *σ* is the standard deviation (STD). An experiment was conducted with *σ* ranging from 0.02 to 0.20 for the chosen range of systolic and diastolic ratios, 0.65 to 0.95, and 0.30 to 0.60, respectively [11]. It was observed that the likelihood function was almost unaffected by changes in *σ* as mentioned in [18].

The likelihoods $f({\hat{y}}_{s(i,j)}|\text{cr}{\left(l\right)}_{s(i,j)})$ of each ratio are the values of the measurement distribution at a measurement value, where ${\hat{y}}_{s(i,j)}$ are the ratios of the pressure values obtained from the reference auscultatory measurement and the maximum amplitude (MA), as given by Equation (15). The goal is to find the SBPR, **cr**(*l*)*_s_*_(_*_i,j_*_)_ that maximizes the likelihood ratio, for the available SBP reference measurements for each subject. The same idea is used for obtaining the DBPR. Since two reference auscultatory measurements are available, the average SBP and DBP measurement is used as the reference to obtain the SBPR and DBPR. We also apply the Laplacian (L) model [14] to obtain the likelihoods of each ratio such that

(13) $$f_{L}({\hat{y}}_{s(i,j)}|\text{cr}{\left(l\right)}_{s(i,j)})=\frac{1}{\sqrt{2\sigma }}\exp^{-\frac{\sqrt{2}}{\sigma }\left|{\hat{y}}_{s(i,j)}-\text{cr}{\left(l\right)}_{s(i,j)}\right|}$$

(14) $$f_{L}({\hat{y}}_{d(i,j)}|\text{cr}{\left(l\right)}_{d(i,j)})=\frac{1}{\sqrt{2\sigma }}\exp^{-\frac{\sqrt{2}}{\sigma }\left|{\hat{y}}_{d(i,j)}-\text{cr}{\left(l\right)}_{d(i,j)}\right|}$$

The reference SBPR and DBPR are obtained for the *j^th^* measurement of the *i^th^* subject as follows:

(15) $${\hat{y}}_{s(i,j)}=\frac{{\hat{a}}_{s(i,j)}}{m_{\left(i,j\right)}}$$

(16) $${\hat{y}}_{d(i,j)}=\frac{{\hat{a}}_{d(i,j)}}{m_{\left(i,j\right)}}$$

where ${\hat{y}}_{s(i,j)}$ and ${\hat{y}}_{d(i,j)}$ denote the reference SBPR and DBPR obtained using the auscultatory nurse measurements, and ${\hat{a}}_{s(i,j)}$ and ${\hat{a}}_{d(i,j)}$ are the amplitudes of the SBP and the DBP which are identified on the oscillometric waveform (OMW)'s envelope through the deflation curve giving auscultatory nurse measurements for the SBP and DBP, respectively. The maximum amplitude *m*_(_*_i,j_*_)_ corresponds to the maximum amplitude in the OMW's envelope.

The following procedure is used for obtaining the SBP and DBP estimates using the conventional approach. The OMW is recovered using the CP and the pulse derivative waveform (PDW) [8] is obtained. The local maxima of the OMW are used in order to build an envelope. The envelope of the OMW is then smoothed using cubic spline interpolation [19] that is commonly used to reduce interference such as movement artifacts from the oscillometric BP envelope [4]. Based on the MA and the CP, SBP and DBP are determined using the conventional experimentally obtained systolic and diastolic ratios [5] which are 0.70 and 0.45, respectively [4].

The SBP and DBP estimates are also obtained using Bayesian inference [18] and then the results of the conventional MAA and the proposed method are compared.

**Figure 4.**
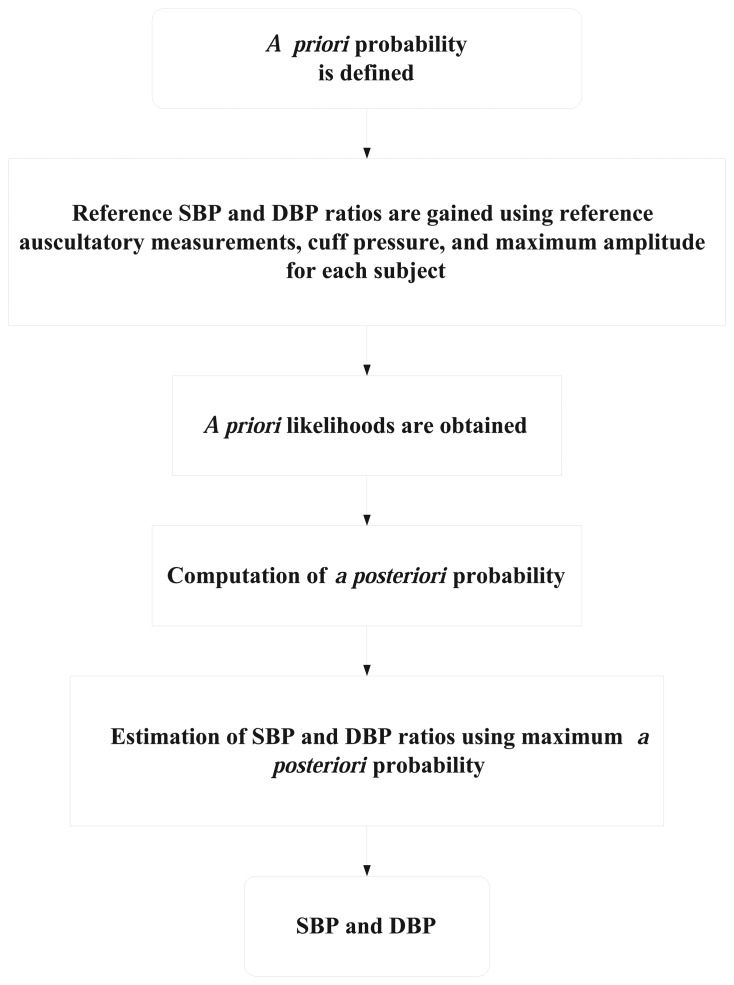
The estimation of SBP and DBP is used to estimate the SBP and DBP ratio based on the Bayesian mode.

The following step by step procedure is used to estimate the SBP and DBP ratios using the Bayesian approach as shown Figure 4.

(1) As the first step, the ranges of the systolic and diastolic ratios used in the proposed method are initially found experimentally [2,3] and the PP is defined as shown in Equations (7) and (8).
(2) The SBP and DBP estimates are obtained using both the MA value and the fixed *a priori* ratios of SBP and DBP
(3) The reference SBPR and DBPR are obtained using the reference auscultatory measurement, which itself is obtained using the cuff pressure, reference auscultatory measurement, and maximum amplitude for each subject.
(4) The *a priori* likelihoods are obtained as shown in Equation (11) to Equation (12).
(5) The calculation of POP is performed to determine the final ratio of SBP and DBP in Equations (9) and (10).
(6) The SBPR and DBPR that produced the maximum *a posteriori* probability in Equation (17) are taken as the best ratio for the measurement. As the estimation of the SBPR and DBPR is based on the *a priori* probability and the likelihood function, the final ratios ${\hat{r}}_{i,j}^{s}$ are presented as the maximum *a posteriori* probability.
  (17) $${\hat{r}}_{i,j}^{s}=\arg\underset{\text{cr}{\left(l\right)}_{s(i,j)}}{\text{max}}p(\text{cr}{\left(l\right)}_{s(i,j)}|{\hat{y}}_{s(i,j)})$$
  Similarly, the ratio for the DBP, ${\hat{r}}_{i,j}^{d}$ can also be obtained. Using these ratios, the SBP and DBP estimates are obtained. In the method above, each measurement will produce one SBPR and DBPR.

## 3. Experimental Results and Discussion

### 3.1. Subjects and Data Collection

The local research ethics committee approved the research, and all subjects provided informed consent prior to the BP measurement according to the protocol of the institutional research ethics board. The oscillometric measurements for this study were provided by Biosign Technologies Inc. (Toronto, Ontario, Canada). The experimental data set was acquired from 85 healthy subjects aged from 12 to 80, out of which thirty seven were females and forty eight were males. No recruited subject had any history of cardiovascular disease. Five sets of oscillometric BP measurements were obtained from each volunteer (5 × 85 = 425 total measurements: duration range to record a single measurement: 31–95 s, duration median: 55 s) using a wrist worn UFIT^®^ blood pressure device [4,20,21] (Biosign Technologies Inc., Toronto, Ontario, Canada) in accordance with the recommendations of the ANSI/AAMI SP 10 standard [15]. Specifically, the two nurse readings are averaged to provide one SBP and one DBP reading as the reference which is an auscultatory method (mmHG) considered the standard protocol for noninvasive BP measurement [15]. The data set provided contains relatively stable nurse readings, in that the maximum difference between the two nurses is no more than 2 mmHG. This again satisfies the recommendations of the ANSI/AAMI SP 10 standard, which requires the mean difference to be no more than 5 mmHG. Nurse reading of SBP ranged from 78 to 147 mmHG and DBP ranged from 42 to 99 mmHG across all subjects. Specifically, our procedure of our BP measurements consists of an oscillometric blood pressure recoding, followed by readings of SBP and DBP with help of two trained nurse after a one minute pause. This is then followed by another one minute pause. The procedure is repeated again four more time to make the recoding of five measurements. For data collection, each subject sat well and upright posture in a chair where the UFIT monitor's cuff is strapped to the left wrist of the subject, which is raised to heart level. Another cuff, which is the component of the auscultatory, is placed on the upper left arm also at heart level.

In this paper, only five measurements of each subject were assumed to be available and were used to implement was used as the true estimate. In Tables 1 and 2, we presents the averaged (over 85 subjects) systolic blood pressure (SBP) and diastolic blood pressure (DBP) estimates for a sequence of five measurements determined by the proposed algorithm as well as the corresponding averaged systolic and diastolic ratios obtained through the proposed method for the five measurements. The standard deviation of the estimates is provided in brackets. Tables 3 and 4 contain the average results obtained using the proposed individualized ratios for 85 subjects using the five measurements. The proposed methods are referred to as the maximum amplitude algorithm using Bayesian with Gaussian (MAABG) and the maximum amplitude algorithm using Bayeaisn with Laplacian (MAABL).

In order to verify the performance of BP estimation, the MD and the SD between the estimated BP and the auscultatory nurse measurements were calculated according to AAMI standard protocol recommended [15]. A blood pressure monitor could pass AAMI protocol, if its measurements error has a mean value of less than 5 mmHG with a SD of no more than 8 mmHG [15] Therefore, lower values of MD correspond to better overall performance. The MD of the proposed MAABG algorithm in SBP and DBP was compared to that of the MAA algorithm as in Table 5, confirming that the proposed MAABG has much effect on the error of the estimate. Tables 5 shows the MD as the average difference between the blood pressures measured through auscultation by the nurse, and the MAA and MAAB methods described in this paper. For example, the MD was found to be 6.80 and 5.39 (mmHG) for the SBP and DBP when comparing MAA and the reference ausculation method based on the result of the first measurement. The proposed method, MAABG showed lower MD compared to the MAA for both SBP and DBP consistently in all the five measurements, as shown in Table 5. In addition, the SD was used to describe a measure of error variability between the auscultatory nurse measurements and the estimates obtained using the proposed method. The SD between the proposed MAABG method and the auscultatory nurse measurements was found to be 2.99 mmHG for the SBP and 3.58 mmHG for the DBP, respectively, which were superior to those obtained from the auscultatory nurse measurements and the MAA method.

**Figure 5.**
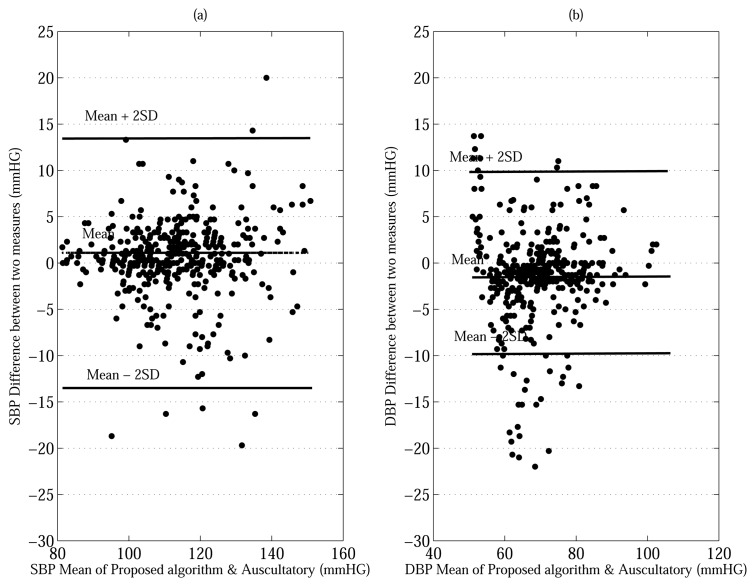
Bland-Altman plots comparing of the performance between the proposed (MAABG) algorithm and auscultatory results, (**a**) Bland-Altman plot for the SBP and (**b**) Bland-Altman plot for the DBP.

**Figure 6.**
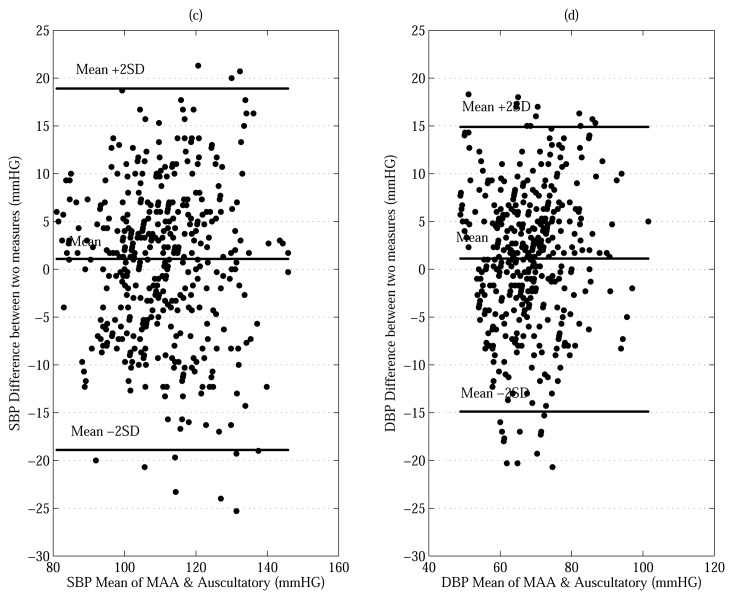
Bland-Altman plots comparing of the performance between thr conventional MAA and auscultatory results, (**a**) Bland-Altman plot for the SBP and (**b**) Bland-Altman plot for the DBP.

The work described in this paper is a systematic methodology with a theoretical basis using a Bayesian model for estimating the SBP and DBP. As seen from in the last row of Table 5, the proposed approaches, evaluated using both the MAABG and MAABL resulted in much lower MD's average for both SBP (MD = 6.25 and 6.25 mmHG) and the DBP (MD = 5.00 and 5.00 mmHG) compared to the conventional MAA. The difference in estimation error between the proposed methods and the conventional MAA for SBP and DBP is an MD of 0.78 and 0.95 mmHG. Figure 5 shows Bland-Altman plots comparing of the performance between the proposed MAABG algorithm and auscultatory nurse measurements (425 measurements) [22]. The performance between the conventional MAA and auscultatory nurse measurements (425 measurements) is compared by Bland-Altman plots as shown in Figure 6. The limits of agreement (see bold horizontal lines in Figures 5 and 6) that we use are (MD ± 2× SD) for all plots. For all plots in Figures 5 and 6, most of BP measurements' points within the limits of agreement. Moreover, the bias (see horizontal center lines) for all plots is negligible amount (< ±1.5 mmHG). This implies that the BP estimates made by the MAA and MAABG are in close agreement with those made by the auscultatory nurse measurement without being overly biased in any particular direction. We also note that the vertical spreads of the proposed MAABG algorithm for the SBP and DBP are smaller than those of the conventional MAA method as shown in Figures 5 and 6. That is, the proposed MAABG algorithm provides an improvement in oscillometric BP estimation.

In addition, the SD was utilized as a tool of error variable between the auscultatory nurse measurements and the estimates obtained using the proposed MAABG. The SD obtained with the proposed methods, which are the MAABG and MAABL, was found to be 3.32 and 3.32 mmHG for the SBP, respectively, and 3.34 and 3.34 mmHG for the DBP, respectively. This performance is superior to that obtained when the conventional MAA was compared with the auscultatory nurse measurements. The difference in SD between the proposed MAABG and the conventional MAA, for SBP and DBP is 2.99 and 1.13 mmHG. Such improvements could be very significant given that the AAMI protocol recommends for the automated BP monitors [15]. These results confirm that the proposed approaches which are based on the Bayeaian model acquires a high degree of accuracy in BP measurements. Note that, the proposed MAABG and MAABL shows the same results. This implies that the proposed approaches represent the robust characteristics despite using different likelihood function.

## 4. Conclusions

In conclusion, the MDs and SDs of the SBP and DBP obtained through the proposed MAABG and MAABL are smaller relative to the reference nurse values when compared to the conventional MAA method. This study has established that the proposed method has outperformed the conventional MAA method in estimating the SBP and DBP. Furthermore, a systematic methodology with a theoretical basis for calculating individualized SBPR and DBPR is demonstrated that can be used with conventional MAA algorithm.

## Figures and Tables

**Table 1.** Ratio averages of systolic blood pressure (SBP) measurements by the maximum amplitude algorithm using Bayesian with Gaussian (MAABG) and the maximum amplitude algorithm using Bayesian with Laplacian (MAABL); *n* = 85 is number of subject; *std* is standard deviation.

**Method** **(n = 85)**	**Ratio (std)** **First**	**Ratio (std)** **Second**	**Ratio (std)** **Third**	**Ratio (std)** **Fourth**	**Ratio (std)** **Fifth**
MAABG	0.79 (0.11)	0.79 (0.12)	0.79 (0.12)	0.80 (0.11)	0.80 (0.11)
MAABL	0.79 (0.11)	0.79 (0.12)	0.79 (0.12)	0.80 (0.11)	0.80 (0.11)

**Table 2.** Ratio averages of diastolic blood pressure (DBP) measurements by the maximum amplitude algorithm using Bayesian with Gaussian (MAABG) and the maximum amplitude algorithm using Bayesian with Laplacian (MAABL); *n* = 85 is number of subject; *std* is standard deviation.

**Method** **(n = 85)**	**Ratio (std)** **First**	**Ratio (std)** **Second**	**Ratio (std)** **Third**	**Ratio (std)** **Fourth**	**Ratio (std)** **Fifth**
MAABG	0.44 (0.11)	0.43 (0.11)	0.42 (0.11)	0.41 (0.11)	0.39 (0.11)
MAABL	0.44 (0.11)	0.43 (0.11)	0.42 (0.11)	0.41 (0.11)	0.39 (0.11)

**Table 3.** Summary of the averaged systolic blood pressure (SBP) estimates by the nurse, the maximum amplitude algorithm (MAA), MAA using Bayesian with Gaussian (MAABG), and the maximum amplitude algorithm (MAA), MAA using Bayesian with Laplacian (MAABL); *std* is standard deviation; first to fifth is the sequence of measurements.

**BP (mmHG)** **(N = 85)**	**SBP (std)** **First**	**SBP (std)** **Second**	**SBP (std)** **Third**	**SBP (std)** **Fourth**	**SBP (std)** **Fifth**
**Nurse**	108.9 (13.2)	108.9 (13.5)	109.8 (13.8)	110.3 (13.5)	112.2 (14.6)
**MAA**	115.4 (14.0)	115.5 (14.4)	116.3 (14.5)	118.1 (14.3)	120.0 (15.4)
**MAABG**	114.8 (13.2)	114.6 (13.3)	115.6 (13.1)	116.7 (13.5)	118.5 (14.6)
**MAABL**	114.8 (13.2)	114.6 (13.3)	115.6 (13.1)	116.7 (13.5)	118.5 (14.6)

**Table 4.** Summary of the averaged diastolic blood pressure (DBP) estimates by the nurse, the maximum amplitude algorithm (MAA), MAA using Bayesian with Gaussian (MAABG), and the maximum amplitude algorithm (MAA), MAA using Bayesian with Laplacian (MAABL); *std* is the standard deviation; first to fifth is the sequence of the measurements.

**BP (mmHG)** **(N = 85)**	**DBP (std)** **First**	**DBP (std)** **Second**	**DBP (std)** **Third**	**DBP (std)** **Fourth**	**DBP (std)** **Fifth**
**Nurse**	67.6 (9.8)	67.1 (9.6)	67.2 (10.0)	67.6 (9.8)	67.5 (10.2)
**MAA**	69.7 (10.5)	69.8 (10.4)	69.9 (10.3)	72.0 (10.5)	73.2 (10.8)
**MAABG**	70.7 (10.3)	70.4 (9.9)	70.7 (9.9)	72.0 (9.9)	71.9 (9.9)
**MAABL**	70.7 (10.3)	70.4 (9.9)	70.7 (9.9)	72.0 (9.9)	71.9 (9.9)

**Table 5.** Summary of the MD and SD obtained using the MAA and the proposed methods (MAABG and MAABL) relative to the reference auscultatory method.

**MAA (mmHG) Test**	**SBP MD (SD)**	**MAABG DBP MD (SD)**	**SBP MD (SD)**	**MAABL DBP MD (SD)**	**SBP MD (SD)**	**DBP MD (SD)**
1st mea.	6.80 (6.70)	5.39 (5.10)	6.01 (2.99)	4.92 (3.58)	6.01 (2.99)	4.92 (3.58)
2nd mea.	7.80 (6.22)	5.60 (4.40)	5.87 (3.27)	4.56 (2.93)	5.87 (3.27)	4.56 (2.93)
3rd mea.	6.70 (5.80)	5.70 (4.20)	6.45 (3.73)	4.88 (3.39)	6.45 (3.73)	4.88 (3.39)
4th mea.	6.30 (5.77)	6.00 (4.09)	6.14 (2.84)	5.01 (3.32)	6.14 (2.84)	5.01 (3.32)
5th mea.	7.56 (7.07)	7.08 (4.98)	6.76 (3.79)	5.63 (3.87)	6.76 (3.79)	5.63 (3.87)

avg.	7.03 (6.31)	5.95 (4.55)	6.25 (3.32)	5.00 (3.42)	6.25 (3.32)	5.00 (3.42)

## Appendix

Here, we add the basic concept of Bayes rule [18] in more detail for the individualized SBPR and DBPR which are already assumed in Section 2.2 as random variables $P({\hat{r}}_{i,j}|h).$ Employing the definition of conditional probability with the product and sum rules produces Bayes rule such that

(18) $$p(\zeta |\eta )=\frac{p(\eta \cap \zeta )}{p(\eta )}=\frac{p(\eta \cap \zeta )}{p(\eta \cap \zeta )+p(\eta \cap \bar{\zeta })}=\frac{p(\zeta )\times p(\eta |\zeta )}{\left\{p(\zeta )\times p(\eta |\zeta )\}+\{p(\bar{\zeta })\times p(\eta |\bar{\zeta })\right\}}$$

where *ζ* and *η* represent independent events. Note that, the *ζ* is a considered unobservable and the *η* is an observable event. The *ζ* is called *a priori* probability. The likelihood function of the unobservable event *ζ* is then given by *p*(*η*∣*ζ*). Thus, *p*(*η*∣*ζ*) is the *posteriori* probability of event *η*. Bayes's rule is actually just a rewriting of the conditional probability equation, where the joint probability in numerator is known as the multiplication rule, and the marginal probability in denominator is known as using the law of total probability followed by the multiplication rule.

Based on Equation (18), we can see the parts in Equation (7). The prior distribution is given by the **pp***_s_* vector in Equation (5) which are assigned to equal candidate ratios due to the lack of any a priori information. We then observe the likelihood function $f({\hat{y}}_{s(i,j)}|\text{cr}{\left(l\right)}_{s(i,j)}).$ This conditional probability function of ${\hat{y}}_{s(i,j)}$ given **cr**(*l*)*_s_*_(_*_i,j_*_)_), actually, the value that occurs and where **cr**(*l*)*_s_*_(_*_i,j_*_)_) is allowed to vary over its whole range for *α*_1_,…, *α_k_*. Thus, the posteriori probability distribution is given by the a POP $p(\text{cr}{\left(l\right)}_{d(i,j)}|{\hat{y}}_{s(i,j)})$ evaluated at **cr**(*l*) for every *l*, *l* = 1, ⋯, *K*. This equation gives us a technique for revising our belief probabilities with respect to the possible values of **cr**(*l*) given that we obtained ${\hat{y}}_{s(i,j)}$) from the reference auscultatory measurement. Consequentially, we obtain the final ratios ${\hat{r}}_{i,j}^{s}$ as given in Equation (15).
